# Dual-energy CT for therapy monitoring: histogram analyses of iodine maps reveal typical pattern of enhancement

**DOI:** 10.1186/1470-7330-14-S1-P17

**Published:** 2014-10-09

**Authors:** M Uhrig, D Simons, M Ganten, J Hassel, HP Schlemmer

**Affiliations:** 1 German Cancer Research Center (DKFZ) Heidelberg, Department of Radiology, Im Neuenheimer Feld 280, D-69120 Heidelberg, Germany; 2 Thoraxklinik at Heidelberg University Hospital, Department of Diagnostic and Interventional, Radiology, Amalienstr. 5, D-69121 Heidelberg, Germany; 3 National Center for Tumour Diseases (NCT) Heidelberg, Im Neuenheimer Feld 460, D-69120 Heidelberg, Germany

## Aim

Functional information for appropriate therapy monitoring of advanced targeted therapies is essential, but standard follow up (FU) examinations with computed tomography (CT) still focus on traditional size measurements. Iodine quantification with dual energy CT (DECT) enables additional quantitative assessment of contrast media uptake. Our purpose was to investigate patterns of contrast media enhancement under BRAF inhibitor therapy (BRAF-I) by performing histogram analyses (HA) of iodine maps based on DECT.

## Methods

11 stage IV melanoma patients underwent DECT at baseline and at least one FU. 8 patients were RECIST-responder to BRAF-I. Volume segmentation of in total 33 metastases was performed semi-automatically. For each lesion, iodine uptake (IU) and HA of iodine maps including maximum HU value (max), mean HU value (mean) and standard deviation (STD) was calculated.

## Results

For BRAF-responder mean, max and STD of the iodine histograms decrease significantly (p<0.05 at FU 2). In patients with progress, 6 of 7 lesions showed increasing max and STD, while mean and IU were decreasing (4 lesions) as well as increasing (3 lesions). Analysis of the metastasis with mixed response revealed about stable values for mean, max and STD for the responding part and increasing values for the viable tumour.

## Conclusion

For patients under BRAF-I, HA of iodine maps based on DECT revealed a typical pattern of contrast media enhancement. HA has potential to add an objective and functional criterion to traditional size measurements of standard CT examinations and can contribute to accurate response assessment for BRAF-I therapy.

